# Supplementary material for: OsNAC15 Regulates Tolerance to Zinc Deficiency and Cadmium by Binding to OsZIP7 and OsZIP10 in Rice
Source: Int J Mol Sci. 2022 Oct 4;23(19):11771. doi: 10.3390/ijms231911771 (PMC9569620; doi:10.3390/ijms231911771)
Supplement: Supplementary file 1 [file ijms-23-11771-s001.zip › Supplementary figures.pdf]

International Journal of *Molecular Sciences*

MDPI

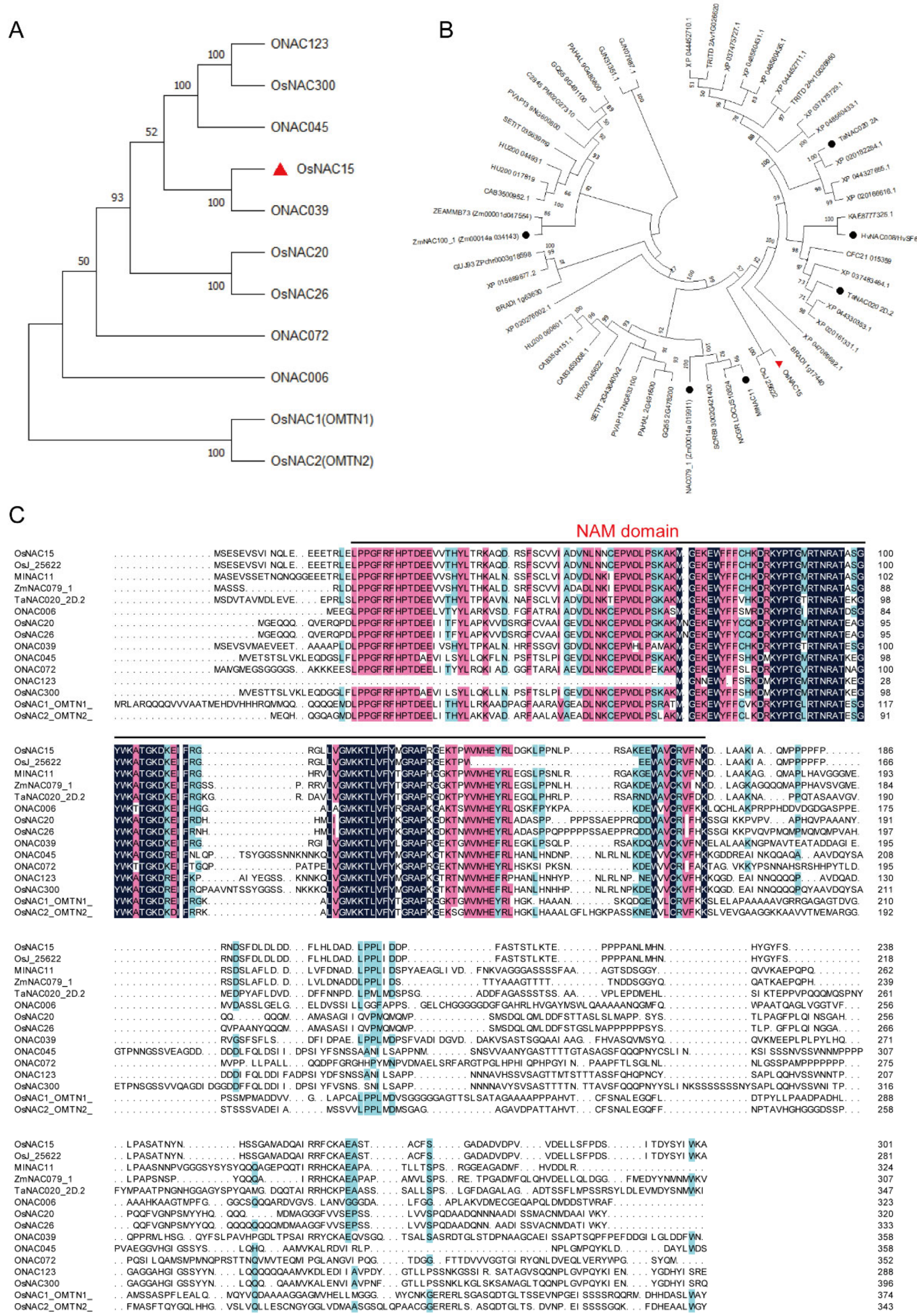

Figure S1. Phylogenetic analysis of OsNAC15. Phylogenetic analysis of OsNAC15 in rice (*Oryza sativa*) (**A**) and other species mainly including *Zea mays*, *Triticum aestivum*, *Hordeum vulgare*, *Miscanthus lutarioriparius*, *Panicum hallii* and *Sorghum bicolor* (**B**). Phylogenetic trees were constructed by neighbor-joining method and bootstrap values from 1000 replicates. The red triangle indicates OsNAC15. The black circles indicate other NAC proteins. Numbers on each branch represent the bootstrap percentage. (**C**) Multiple sequence alignment of NAC proteins. Sequence under the black bar represent the NAM domain.

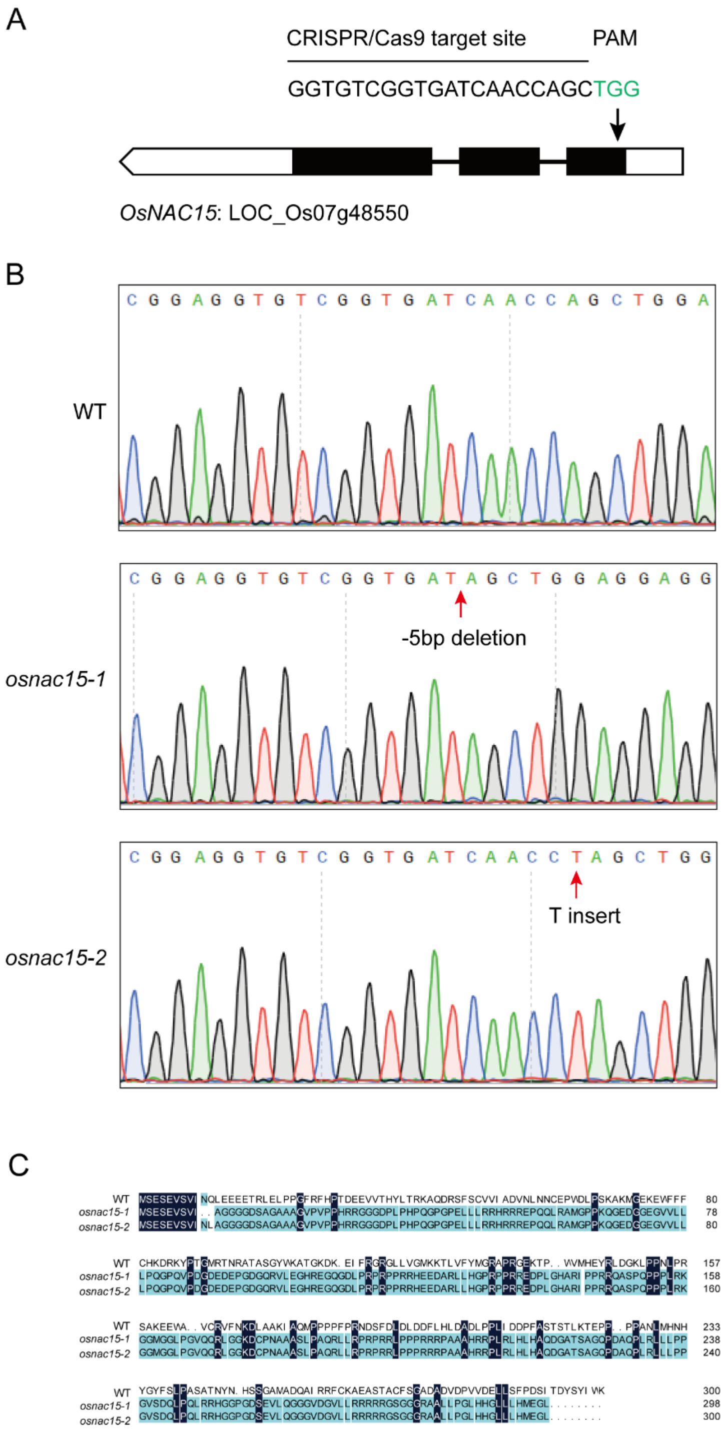

Figure S2. Molecular characterization of *osnac15* mutants. (**A**) Schematic presentation of the gene structure of *OsNAC15* and CRISPR/Cas9 editing site. Black boxes: exons. PAM: protospacer adjacent motif. Top part: Sanger sequencing chromatograph of the CRISPR/Cas9 target site in the homozygous mutants of *osnac15*. The letter in green represented the PAM sequence. (**B**) The specific deletion and insertion of bases in the *osnac15* mutant lines are indicated by red arrows. (**C**) Protein sequence alignment of OsNAC15 in the WT and *osnac15* mutant lines.
