# Supplementary figures and images for: OsNAC15 Regulates Tolerance to Zinc Deficiency and Cadmium by Binding to OsZIP7 and OsZIP10 in Rice
Source: Int J Mol Sci. 2022 Oct 4;23(19):11771. doi: 10.3390/ijms231911771 (PMC9569620; doi:10.3390/ijms231911771)

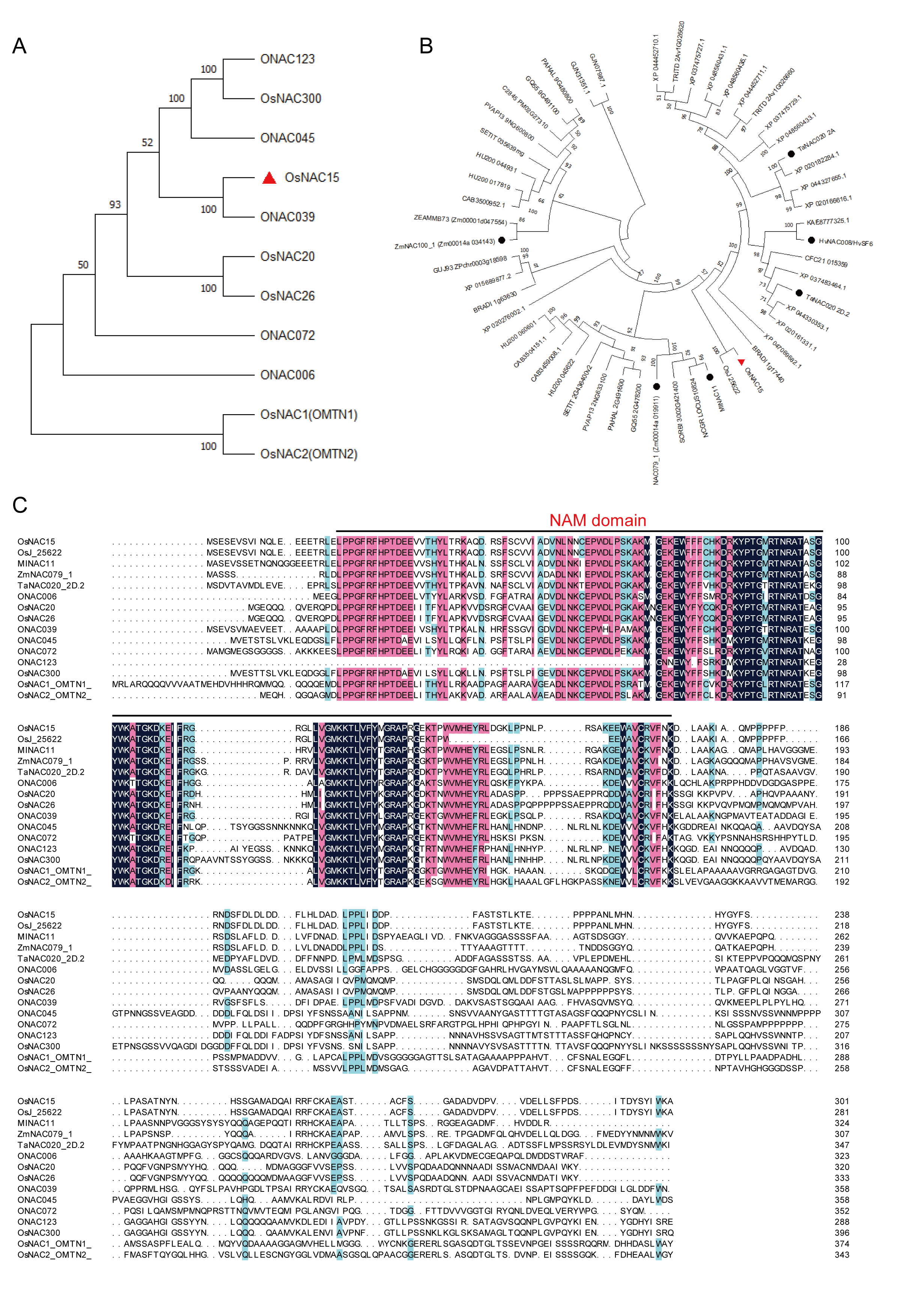

Supplement: Supplementary file 1 [file ijms-23-11771-s001.zip › Figure S1.tif]

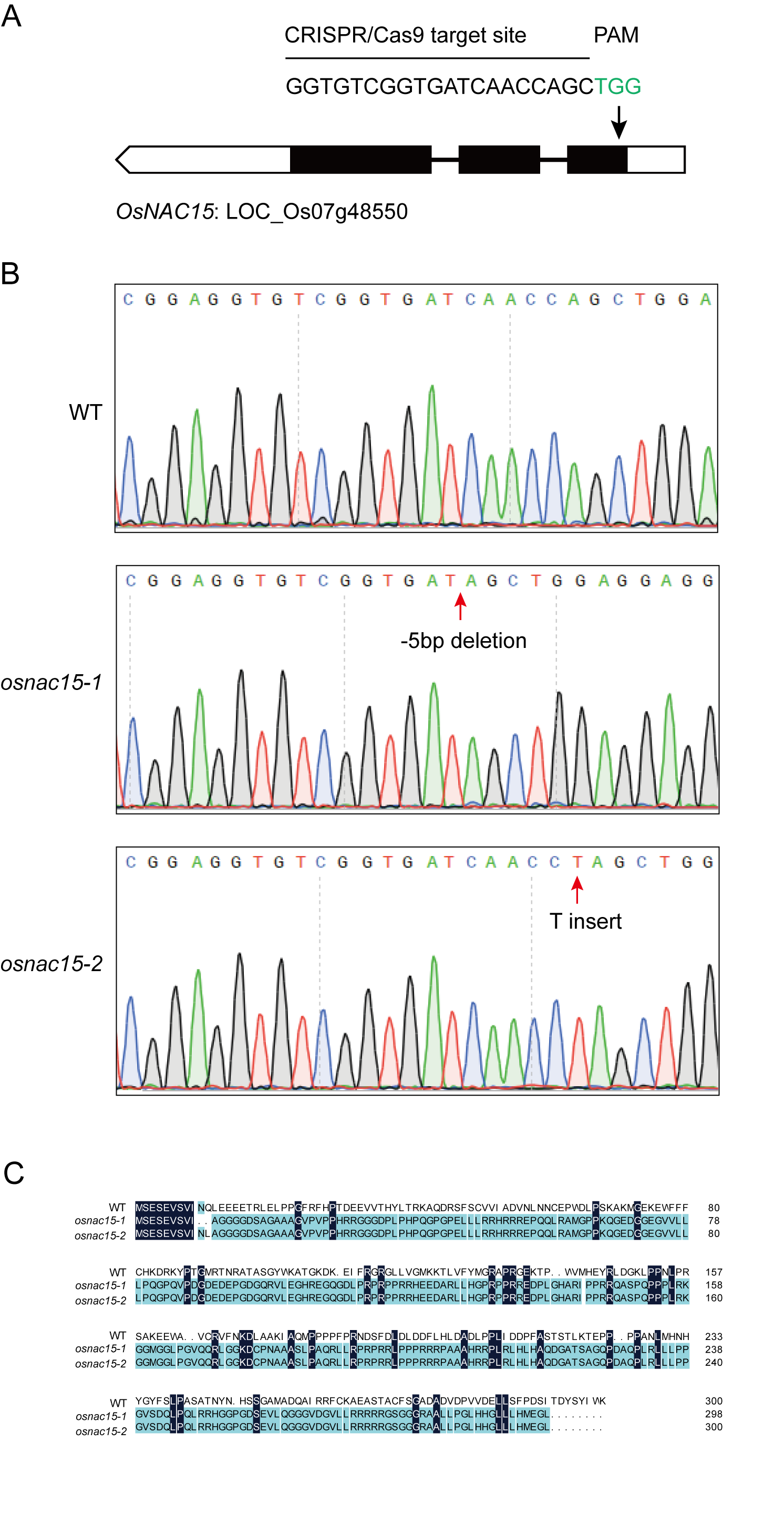

Supplement: Supplementary file 1 [file ijms-23-11771-s001.zip › Figure S2.tif]
